# Pelvic sleeve pyeloplasty in adults: A technical refinement of Anderson–Hynes dismembered pyeloplasty

**DOI:** 10.1002/bco2.70301

**Published:** 2026-11-02

**Authors:** Dinesh K. Agarwal

**Affiliations:** ^1^ Department of Surgery, Melbourne Medical School The University of Melbourne Melbourne Victoria Australia; ^2^ Department of Urology Western Health Melbourne Victoria Australia; ^3^ Department of Urology The Royal Melbourne Hospital Melbourne Victoria Australia

**Keywords:** hydronephrosis, laparoscopy, pyeloplasty, robotic surgical procedures, ureteral obstruction

## Abstract

**Objectives:**

To describe pelvic sleeve pyeloplasty, a technical refinement of Anderson–Hynes dismembered pyeloplasty for adult ureteropelvic junction obstruction, and to report perioperative and functional outcomes in an initial adult series.

**Methods:**

Between 2009 and 2018, 12 adult patients with symptomatic ureteropelvic junction obstruction underwent pelvic sleeve pyeloplasty using a transperitoneal laparoscopic or robot‐assisted approach. Preoperative evaluation included computed tomography intravenous pyelography and diuretic Tc‐99m MAG3 renography. Patients with previous failed ureteropelvic junction surgery were excluded. The technique preserves and incorporates a sleeve of renal pelvic tissue into the reconstruction to create a broad, continuous, tension‐free ureteropelvic anastomosis while minimizing manipulation of healthy ureteric tissue.

**Results:**

All procedures were completed successfully without conversion to open surgery. Eight patients underwent laparoscopic and four underwent robot‐assisted pyeloplasty. Mean operative time was 185 min (range 160–255 min), and estimated blood loss ranged from 90 to 350 mL. One patient had intraoperative bleeding from a gonadal vein injury, controlled without transfusion. One patient developed a transient postoperative febrile episode (Clavien–Dindo grade II). No patient developed urinary leakage or required secondary intervention. At a mean follow‐up of 38 months (range 24–50 months), all patients were asymptomatic and diuretic Tc‐99m MAG3 renography demonstrated unobstructed drainage.

**Conclusions:**

Pelvic sleeve pyeloplasty is a feasible modification of dismembered pyeloplasty that preserves ureteric length, minimizes ureteric manipulation and facilitates a wide, continuous, tension‐free ureteropelvic anastomosis. Larger comparative studies are required to validate reproducibility and long‐term efficacy.

## 1 INTRODUCTION

Ureteropelvic junction obstruction (UPJO) is characterized by functionally significant impairment of urine transport from the renal pelvis to the proximal ureter, leading to hydronephrosis and potential deterioration of renal function. Although often congenital, UPJO may present in adulthood with flank pain, urinary tract infection, nephrolithiasis or declining renal function. The aims of treatment are durable relief of obstruction, symptom resolution and preservation of renal function.

Anderson–Hynes dismembered pyeloplasty remains the reference standard surgical treatment for UPJO and may be performed using open, laparoscopic or robot‐assisted approaches.^1^ Since its original description in 1949, the technique has undergone several refinements, but its core principles remain excision of the stenotic ureteropelvic segment, ureteric spatulation and construction of a dependent, tension‐free ureteropelvic anastomosis.^2^, ^3^

Despite excellent reported outcomes, conventional dismembered pyeloplasty has potential technical limitations in selected cases. Excision of the narrowed ureteropelvic segment may result in relative ureteric shortening, particularly when the narrowed segment is long. Ureteric spatulation is often limited to approximately 1–1.5 cm and may not always provide a generous anastomotic circumference. Urinary leakage, recurrent obstruction and late failures remain recognized complications after laparoscopic pyeloplasty.^4^, ^5^ Repeated manipulation of the spatulated ureteric edge during reconstruction may also compromise tissue integrity and vascularity.

We describe pelvic sleeve pyeloplasty (PSP), a technical modification of dismembered pyeloplasty designed to preserve ureteric length, create a wide continuous ureteropelvic anastomosis and minimize manipulation of healthy ureteric tissue. This study presents the surgical technique and perioperative and mid‐term functional outcomes in an initial adult series.

## 2 METHODS

Between 2009 and 2018, 12 adult patients underwent PSP for symptomatic UPJO. All patients were male, with a mean age of 34 years (range 23–48 years). The procedure was performed on the right side in five patients and on the left side in seven patients. All patients presented with recurrent flank pain. Preoperative computed tomography intravenous pyelography demonstrated features suggestive of UPJO, which was confirmed by diuretic Tc‐99m MAG3 renography. Patients with previous failed ureteropelvic junction surgery were excluded. No patient underwent preoperative ureteric stent placement. All procedures were performed transperitoneally.

All patients were catheterized with an indwelling urethral catheter before surgery. Procedures were performed under general anaesthesia by an experienced reconstructive urologist. Eight patients underwent conventional laparoscopic pyeloplasty and four underwent robot‐assisted laparoscopic pyeloplasty using standard port configurations.

After exposure of the renal pelvis and proximal ureter, the ureteropelvic junction was identified. The ureteropelvic junction was detached with a sleeve of renal pelvic tissue using an oblique pelvic incision (Figure 1). The ureter was incised laterally across the narrowed segment and extended distally for approximately 1 cm to expose healthy ureteric lumen. Minimal ureteric mobilization was performed to avoid unnecessary tissue handling. When the renal pelvis was capacious or redundant, excess pelvic tissue was excised to tailor the reconstruction. Anterior crossing vessels were identified in four patients, and the reconstruction was transposed anterior to the vessels.

**FIGURE 1:**
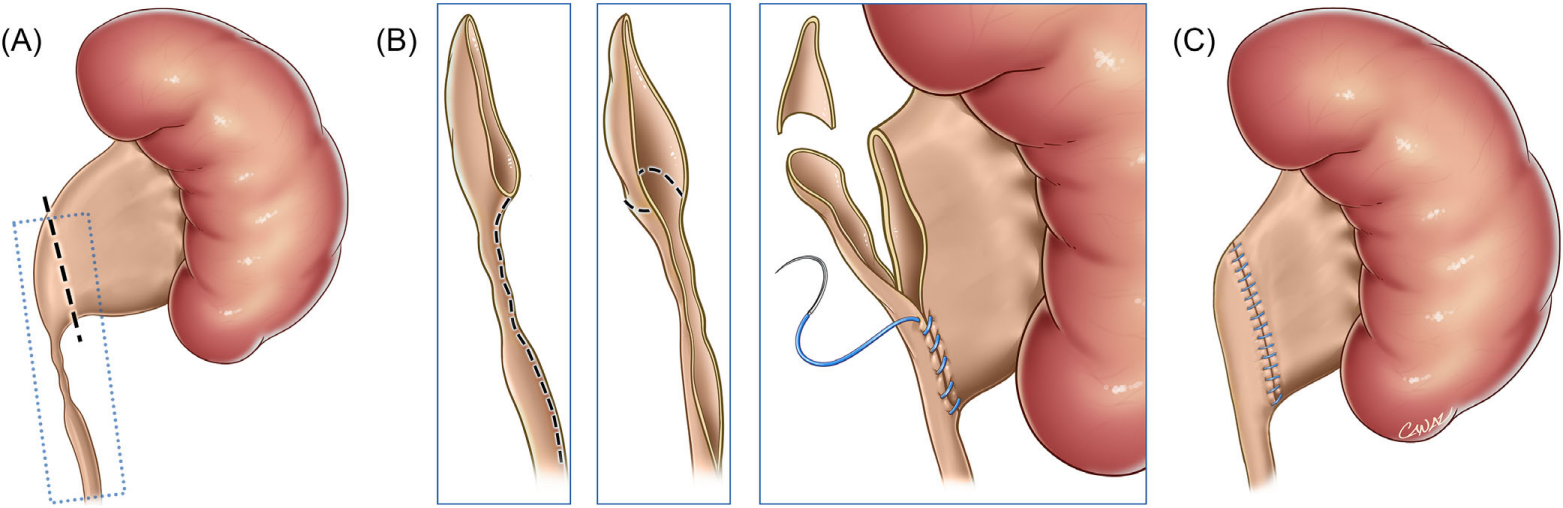
Schematic illustration of pelvic sleeve pyeloplasty. (A) Ureteropelvic junction obstruction with planned oblique pelvic incision. The ureteropelvic junction is detached with a sleeve of renal pelvic tissue. (B) The narrowed ureteropelvic junction is opened longitudinally and extended distally into healthy ureter to create a spatulated ureteric lumen. The retained renal pelvic sleeve is preserved and incorporated into the reconstruction. Reconstruction is commenced at the apex of the spatulated ureter. The pelvic sleeve is anastomosed to the spatulated ureter to create a broad funnel‐shaped ureteropelvic anastomosis. The redundant proximal portion of the pelvic sleeve is excised at completion of the anastomosis. (C) Completed reconstruction demonstrating a wide, dependent, tension‐free ureteropelvic anastomosis with preservation of ureteric length.

Reconstruction commenced at the apex of the spatulated ureter. A 4–0 polyglactin suture was placed through the spatulated ureteric angle and anchored to the dependent portion of the renal pelvic sleeve, securing alignment. The posterior and anterior walls of the pelvic sleeve were then anastomosed to the ureter using continuous 4‐0 polyglactin suture, creating a broad funnel‐shaped, tension‐free ureteropelvic anastomosis. This creates a continuous pelvic sleeve‐ureteric anastomosis rather than a separate pelvic closure line above a shorter ureteropelvic anastomosis. At completion, redundant proximal pelvic sleeve tissue overlying the ureteric tissue was excised. A double‐J ureteric stent was placed intraoperatively in all patients and left in situ for 4 weeks. A closed‐suction drain was placed adjacent to the anastomosis.

The surgical drain was removed on Postoperative Day 1 after confirmation of minimal output. The indwelling urethral catheter was removed on Postoperative Day 2, and patients were discharged when clinically stable. Follow‐up included clinical assessment, urine microscopy and culture, serum renal function testing and diuretic Tc‐99m MAG3 renography at 3 months, 12 months and annually thereafter.

## 3 RESULTS

All 12 procedures were completed successfully using the planned approach without conversion to open surgery. Eight procedures were performed laparoscopically and four using robot assistance. Mean operative time was 185 min (range 160–255 min). Estimated blood loss ranged from 90 to 350 mL. Patient characteristics and perioperative outcomes are summarized in Table 1.

**TABLE 1:** Patient characteristics and outcomes.

Variable	Value
Number of patients	12
Mean age	34 years
Right/left side	5/7
Laparoscopic/robot‐assisted	8/4
Crossing vessels	4
Mean operative time	185 min (range 160–255)
Estimated blood loss	90–350 mL
Length of stay	2 days (one patient 3 days)
Mean follow‐up	38 months (range 24–50)
Success rate	100% (12/12)

One patient experienced intraoperative bleeding secondary to gonadal vein injury, which was controlled without blood transfusion. No intraoperative complication required conversion or procedure abandonment. One patient developed a postoperative febrile episode lasting less than 24 h, which resolved with antibiotics and was classified as a Clavien–Dindo grade II complication.

There was no excessive drain output and no postoperative urinary leak. Surgical drains were removed on Postoperative Day 1 in all cases, and indwelling urethral catheters were removed on Postoperative Day 2. All patients were discharged on Postoperative Day 2, except one patient who was discharged on Postoperative Day 3 because of increased postoperative pain.

Double‐J ureteric stents were removed in all patients 4 weeks after surgery under local anaesthesia using a flexible cystoscope. One patient developed transient flank pain after stent removal, which resolved with conservative treatment and was classified as Clavien–Dindo grade I. No patient required secondary intervention.

At a mean follow‐up of 38 months (range 24–50 months), all patients were clinically asymptomatic. Diuretic Tc‐99m MAG3 renography demonstrated unobstructed drainage in all cases, with no evidence of functional obstruction. All patients met clinical and functional criteria for success.

## 4 DISCUSSION

Anderson–Hynes dismembered pyeloplasty remains the gold standard surgical treatment for UPJO, with reported long‐term success rates of 80% to 98%.^1^ However, Adam and Smith highlighted that Anderson–Hynes pyeloplasty is not uniformly performed as originally described, with variation in renal pelvic incision patterns across published series.^3^ In this context, PSP should be viewed as a technical refinement of dismembered pyeloplasty rather than a departure from its established reconstructive principles.

PSP adheres to the fundamental principles of dismembered pyeloplasty, including dependent drainage, ureteric spatulation, transposition anterior to crossing vessels when indicated, excision of redundant renal pelvis when required and creation of a tension‐free ureteropelvic anastomosis. The principal distinction is that the narrowed ureteropelvic segment is opened longitudinally and incorporated into the reconstruction, whereas conventional Anderson–Hynes pyeloplasty involves complete excision of the stenotic segment.

Several modifications of dismembered pyeloplasty have previously been described to improve tissue handling and orientation. Neulander et al. described a renal pelvic flap that remained attached to the ureteropelvic junction and ureter during reconstruction and served as a no‐touch handle and guide for ureteric spatulation; the flap was discarded before completion of anastomosis.^6^ Ahlawat et al. described post‐anastomotic dismemberment pyeloplasty, in which ureteropelvic continuity was temporarily maintained during suturing to improve orientation before excision of the stenotic segment and redundant pelvis.^7^ PSP differs from these approaches because the opened ureteropelvic junction and retained pelvic tissue are incorporated into the final reconstruction rather than used only as temporary aids.

From a reconstructive perspective, incorporation of the opened ureteropelvic junction increases the amount of tissue available for reconstruction. Successful drainage after pyeloplasty depends on creation of a broad, dependent ureteropelvic junction using healthy, well‐vascularised tissue rather than on anastomotic length per se. In PSP, the opened ureteropelvic junction and adjacent pelvic tissue are incorporated into a broad, funnel‐shaped transition between the renal pelvis and ureter. Importantly, the stenotic segment is not preserved as a narrow tubular conduit; rather, it contributes to the upper part of the reconstruction while the dependent drainage pathway is created through the spatulated healthy ureter and adjacent renal pelvic tissue (Figure 2).

**FIGURE 2:**
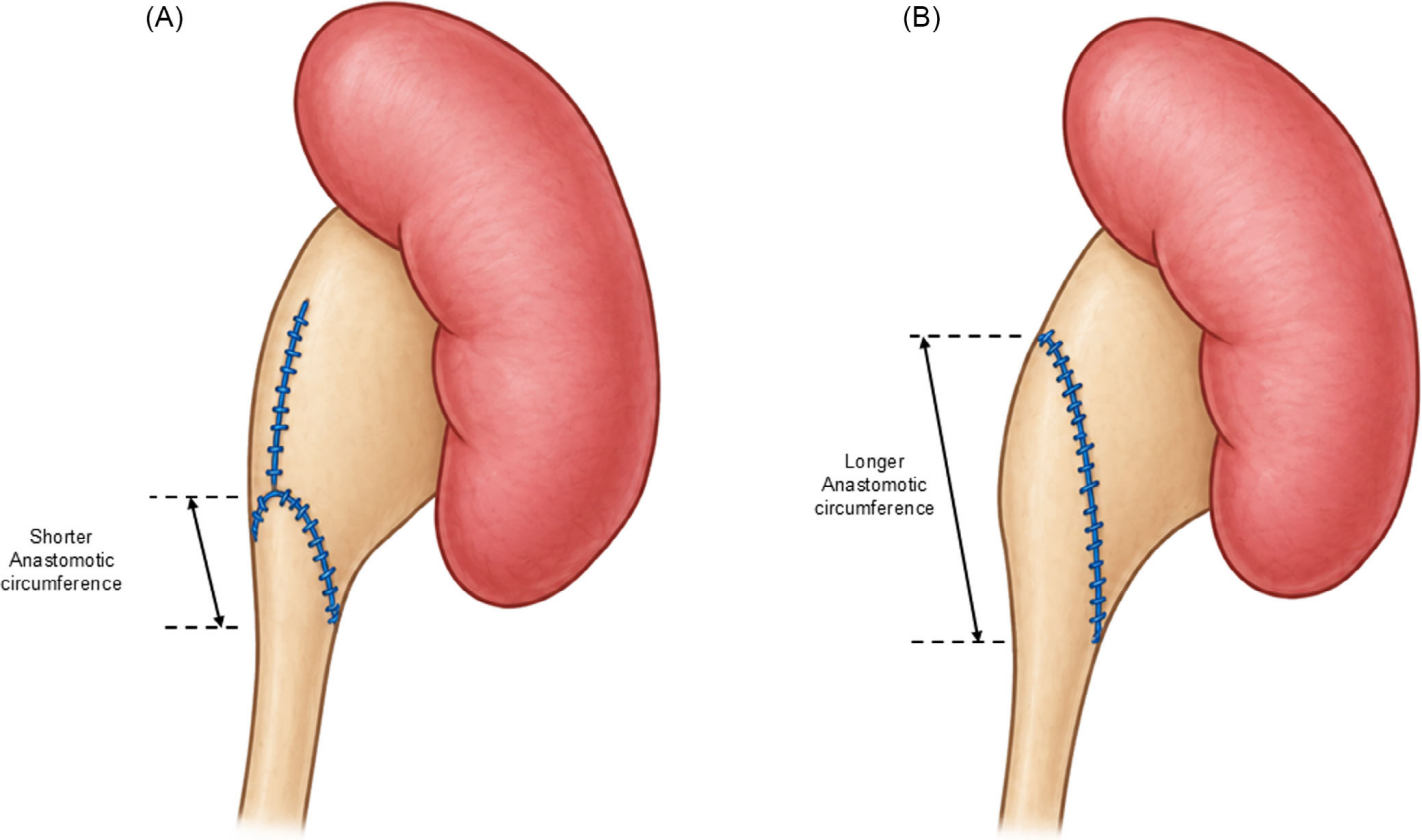
Schematic comparison of anastomotic configuration in reduction Anderson–Hynes pyeloplasty and pelvic sleeve pyeloplasty. (A) In reduction Anderson–Hynes pyeloplasty, after excision of the ureteropelvic junction and redundant renal pelvis, the spatulated ureter is anastomosed to the dependent renal pelvis while the residual renal pelvis is closed separately above the anastomosis. This creates a junction between the pelvic closure line and the ureteropelvic anastomosis. (B) In pelvic sleeve pyeloplasty, the retained pelvic sleeve is incorporated into the ureteropelvic reconstruction, creating a broad, continuous, funnel‐shaped ureteropelvic reconstruction without a separate pelvic closure‐anastomotic junction.

A further theoretical distinction relates to the configuration of the suture lines. In conventional reduction Anderson–Hynes pyeloplasty, the spatulated ureter is anastomosed to the dependent portion of the renal pelvis, while the remaining redundant pelvis is closed separately. This creates a junction where the ureteropelvic anastomosis meets the separate pelvic closure line, which may be viewed schematically as an inverted Y‐shaped configuration. Although the clinical significance of this junction has not been specifically studied and the precise site of urinary leakage is rarely documented in clinical series, urinary leakage remains a recognized complication of laparoscopic pyeloplasty.^4^, ^5^ It is therefore conceivable that convergence of separate suture lines could represent a potential point of focal weakness or imperfect watertight closure in selected cases. PSP avoids this separate pelvic closure‐anastomotic junction by incorporating the pelvic sleeve directly into a continuous anastomotic line (Figure 2). This proposed advantage remains hypothetical and requires evaluation in larger comparative studies.

This configuration may offer several practical advantages. Preservation of ureteric length may reduce anastomotic tension, particularly in cases of long‐segment narrowing or anatomically challenging reconstruction. The retained pelvic tissue also provides a stable handle during suturing, reducing repeated manipulation of the healthy spatulated ureteric edge and potentially minimizing tissue trauma. In addition, the configuration helps maintain medial and lateral ureteric orientation, facilitating precise alignment and reducing the risk of rotational error.

In this initial series, PSP was successfully performed using both laparoscopic and robot‐assisted approaches. No procedure required conversion to open surgery, no urinary leak occurred and no patient required secondary intervention. At a mean follow‐up of 38 months, all patients were asymptomatic and demonstrated unobstructed drainage on diuretic Tc‐99m MAG3 renography. Although the present series is small, these findings suggest that the technique is reproducible and capable of achieving satisfactory mid‐term functional outcomes.

The present study has several limitations. It represents a small retrospective single‐surgeon experience without a comparative control group. The inclusion of both laparoscopic and robot‐assisted procedures may introduce technical heterogeneity. The absence of histological assessment of the incorporated ureteropelvic junction tissue precludes further evaluation of its pathological characteristics. Larger prospective studies with longer follow‐up and direct comparison with conventional Anderson–Hynes pyeloplasty are required to define reproducibility, safety and long‐term efficacy.

## 5 CONCLUSION

PSP is a feasible modification of Anderson–Hynes dismembered pyeloplasty that preserves ureteric length, minimizes ureteric manipulation and facilitates a wide, continuous, tension‐free ureteropelvic anastomosis. In this initial adult series, the technique was associated with favourable mid‐term clinical and functional outcomes. Larger prospective comparative studies are required to validate its reproducibility, safety and long‐term efficacy.

## AUTHOR CONTRIBUTIONS

**Dinesh K. Agarwal:** Conceptualization; methodology; investigation; data curation; formal analysis; project administration; visualization; writing—original draft; writing—review and editing.

## CONSENT

Written informed consent was obtained from all patients for the surgical procedure. All data were retrospectively reviewed and anonymized, and no patient‐identifiable information is included in this manuscript.

## CONFLICT OF INTEREST STATEMENT

The author declares no conflict of interest.

## Data Availability

The data supporting the findings of this study are available from the corresponding author upon reasonable request, subject to institutional ethics and privacy requirements.
